# The Inhibitory Effect of Toosendanin on the Growth and Development of *Spodoptera litura*

**DOI:** 10.3390/insects17070732

**Published:** 2026-07-16

**Authors:** Wei Lu, Jianhao Dong, Yuhui Xu, GenLin Mao

**Affiliations:** 1 Key Laboratory of the Pest Monitoring and Safety Control of Crops and Forests of the Universities of the Xinjiang Uygur Autonomous Region, College of Agronomy, Xinjiang Agricultural University, Urumqi 830052, China; dong371014@outlook.com (J.D.); x1635181866@163.com (Y.X.); 2 Engineering Research Centre of Cotton, Ministry of Education, Urumqi 830052, China; 3 Institute of Fruit Tree Research, Guangdong Academy of Agricultural Sciences, Guangdong Provincial Key Laboratory of Fruit Tree Science and Technology, Guangzhou 510640, China; maogenlin@163.com; 4 Key Laboratory of South Subtropical Fruit Biology and Genetic Resource Utilization, Ministry of Agriculture and Rural Affairs, Guangzhou 510640, China

**Keywords:** feeding preference, growth and development, nutritional efficiency, toosendanin, *Spodoptera litura*

## Abstract

This study aimed to investigate the inhibitory effect of toosendanin (TSN) on the growth and development of *Spodoptera litura* (Fabricius, 1775), especially in terms of feeding preference, larval growth and development, nutritional indices, and enzyme activities. The feeding preference of *S. litura*, larval growth and development, and nutritional indices were evaluated using the diet incorporation method. The activities of three detoxification, three digestive, and three antioxidant enzymes were determined using spectrophotometric, micro-assay, and microplate methods. The feeding preference of *S. litura* exposed to TSN indicated that the larvae had a significant preference for the untreated (control) feed compared with the TSN-supplemented feed. The growth and development results showed a prolongation of the larval developmental period by approximately one instar in *S. litura* larvae fed TSN-supplemented feed. At the highest concentration (100 μg/g), larval weight was 0.76 times that of the control group, with a 27.34% reduction in the pupation rate and a 23.58% reduction in the emergence rate. All differences were statistically significant compared with the control group. In addition, the pupal and adult deformity rates were significantly higher than those in the control group, being 14.68 and 5.67 times higher, respectively, at this concentration. Growth inhibition increased with increasing TSN concentration. In terms of nutritional efficiency, TSN treatment reduced food intake and decreased food conversion rate, thereby inhibiting the relative growth rate of the 4th instar larvae. Further, TSN treatment significantly increased the activities of trypsin, α-amylase, glutathione S-transferase, carboxylesterase, superoxide dismutase, peroxidase, and catalase, and had no significant effect on lipase activity, and significantly reduced cytochrome P450 enzyme activity. TSN can significantly inhibit the digestion and absorption of nutrients in *S. litura* larvae, slow down their growth and development, and increase the pupal and adult deformity rates, thereby protecting plants against infestation by *S. litura*.

## Simple Summary

*Spodoptera litura* is an agricultural pest that feeds on many important crops and causes serious losses. The use of chemical pesticides remains the primary strategy for controlling this pest. However, the intensive and long-term use of these pesticides has resulted in insect resistance, environmental pollution, and negative impacts on beneficial organisms. Botanical pesticides are considered promising alternatives because they originate from natural sources, break down easily, and are relatively safer for nontarget organisms and ecosystems. Among these, toosendanin (TSN) is an environmentally friendly botanical insecticide used to control lepidopteran pests such as armyworms, cabbage caterpillars, diamondback moths, and tea geometrids. However, its effects on the growth and development of *S. litura* remain inadequately understood. The effects of TSN on the feeding behavior, growth, development, and enzyme activities of *S. litura* were examined in this study. The results showed that TSN significantly influenced food utilization, conversion efficiency, and the activities of digestive, detoxification, and antioxidant enzymes in *S. litura*. It also adversely impacted larval growth, pupation, and adult emergence. These findings suggest that TSN may serve as an effective botanical pesticide for the sustainable management of *S. litura* and contribute to safer pest management by reducing reliance on conventional chemical pesticides in agriculture.

## 1. Introduction

*Spodoptera litura* (Fabricius, 1775) is a highly polyphagous pest infesting more than 300 plant species belonging to 90 families, including economically important crops such as cotton, chili, soybean, and vegetables [1,2,3,4]. The larvae exhibit gregarious feeding behavior, initially clustering on the undersides of leaf and skeletonizing foliage, progressing to complete defoliation during severe outbreaks [5,6]. *S. litura* is widely distributed throughout the Asia–Pacific region, with varying infestation intensities [7]. Its voracious larval feeding causes substantial yield losses, reaching up to 75% in pepper and 13.9–24.7% in soybean production [8,9]. *Spodoptera litura* is one of the most destructive lepidopteran pests in tropical and subtropical regions, damaging vegetable and cotton crops, with annual yield losses ranging from 10% to 30% [10,11].

The use of chemical insecticides remains the most widely used strategy for the rapid control of *S. litura* outbreaks. Conventional insecticides, such as organophosphates, carbamates, and pyrethroids [12], as well as newer insecticides, including spinosad, indoxacarb, emamectin benzoate, chlorantraniliprole, and cyantraniliprole, have been extensively used to control this pest [13]. However, long-term and repeated use of insecticides with similar modes of action has imposed strong selection pressure on field populations of *S. litura*, leading to resistance to both conventional and newer insecticides [13,14]. This development of resistance is mainly associated with enhanced metabolic detoxification mediated by cytochrome P450 (CYP450) monooxygenases, carboxyl/cholinesterases, and glutathione S-transferases, together with reduced target-site sensitivity and other adaptive mechanisms [15]. These resistance issues reduce the sustainability of chemical control and highlight the need to explore alternative or complementary control agents, including plant-derived bioactive compounds such as toosendanin (TSN) [16,17].

TSN is a natural tetracyclic triterpene compound extracted from the bark and fruits of the *Melia toosendan* (Siebold & Zucc.) and *M. azedarach* (L.) trees [18]. TSN exhibits broad-spectrum antifeedant and insecticidal activities against various agricultural pests, including the sweet potato weevil, *Cylas formicarius* (Fabricius, 1798) (Coleoptera: Brentidae), the fall armyworm, *Spodoptera frugiperda* (J.E. Smith, 1797) (Lepidoptera: Noctuidae), and *Tribolium castaneum* (Herbst, 1797) [19,20,21]. A previous study examined the toxicity of TSN in rotifers and found that sublethal concentrations of 1.76–2.59 mg/L significantly reduced population density by 70.8% after 48 h, whereas the surviving individuals exhibited reduced body length, width, and volume [22]. TSN is generally considered an environmentally compatible botanical pesticide with favorable selectivity for nontarget organisms [23].

Besides its insecticidal and antifeedant activities, TSN impacts multiple physiological targets in insects. It has been characterized as a selective presynaptic blocker that interferes with neurotransmitter release. It initially facilitates but subsequently depresses synaptic transmission at neuromuscular junctions and central synapses, providing a mechanistic basis for the paralysis-like symptoms observed in intoxicated insects [24]. In addition, TSN interferes with the endocrine regulation of development. Transcriptomic analysis of *S. frugiperda* showed modulation of genes in the juvenile hormone and ecdysone pathways, accounting for the observed molting abnormalities and reduced pupal weight [25,26]. Furthermore, TSN acts as a feeding deterrent through peripheral gustatory mechanisms. Electrophysiological studies in *Pieris brassicae* (Linnaeus, 1758) (Lepidoptera: Pieridae) larvae demonstrated that TSN stimulated deterrent receptor cells while inhibiting sugar- and glucosinolate-sensitive receptor cells in the maxillary sensilla [27].

Moreover, TSN causes significant damage to the midgut epithelium, including microvillar degeneration and altered enzyme activities, as documented in *Mythimna separata* (Walker, 1865) (Lepidoptera: Noctuidae) and *S. frugiperda* [28,29]. A recent study on *S. litura* showed that TSN induced pathological changes in the midgut, inhibited the activities of acetyl-CoA carboxylase, lipase, α-amylase, and trypsin, and downregulated the expression of genes involved in lipid, protein, and carbohydrate metabolism [30]. Collectively, these findings suggest that TSN suppresses larval growth through multiple mechanisms, including neurotoxicity, feeding deterrence, endocrine disruption, and midgut metabolic dysfunction. Therefore, it shows promise as a plant-derived insecticide for pest management.

Despite these advances, the specific impacts of TSN on *S. litura* remain poorly understood. Therefore, the present study aimed to: (1) evaluate the feeding deterrent effect of TSN on *S. litura* larvae using a two-choice bioassay; (2) quantify the impact of various concentrations of dietary TSN on key parameters, including larval growth, pupation rate, and adult emergence; (3) determine the effects of TSN on nutritional physiology by measuring digestive and conversion efficiencies; and (4) investigate the underlying biochemical mechanisms by assessing the activities of digestive, detoxification, and antioxidant enzymes in the midgut.

## 2. Materials and Methods

### 2.1. Test Insect Source

The *S. litura* used in this study was provided by the Fruit Physiology and Postharvest Research Laboratory of the Fruit Tree Research Institute, Guangdong Academy of Agricultural Sciences. The larvae were reared continuously on an artificial diet in an incubator maintained at a constant temperature of 26 ± 1 °C, a relative humidity of 70 ± 10%, and a 14 h/10 h light/dark photoperiod, without any insecticide exposure throughout the process.

Pupae were kept in insect-rearing containers until adult emergence, and the moths were then supplied with a 10% honey solution absorbed on cotton balls as food. The cotton balls were replaced daily to ensure sufficient nutrition and promote oviposition. White A4 paper sheets were attached to the inner walls of the cages to serve as oviposition substrates. The egg masses were collected daily, and the paper was replaced accordingly. The collected egg masses were placed in insect-rearing dishes lined with artificial diet for hatching. The colony was maintained for breeding in the artificial climate chamber following the aforementioned procedure. All materials and tools used for insect rearing were carefully sterilized and disinfected to ensure a clean rearing environment.

The artificial diet for *S. litura* larvae was purchased from Keyun Biotechnology Co., Ltd. (Jiyuan, China), and was used for the continuous rearing of test insects under laboratory conditions.

### 2.2. Experimental Reagents

TSN (purity ≥ 98%) was purchased from Weikeqi Biotechnology Co., Ltd. (Chengdu, China); dimethyl sulfoxide (DMSO, purity ≥ 99.8%) was obtained from Guangzhou Qixiang Biotechnology Co., Ltd. (Guangzhou, China); and Tween-80 (A100442-0500) was supplied by Shanghai Boglong Biotechnology Co., Ltd. (Shanghai, China).

The soluble total protein (STP) assay kit (ml016897), α-amylase (α-AL) assay kit (ml076677), lipase (LPS) activity assay kit (ml016888), trypsin activity assay kit, hydrogen peroxide (H_2_O_2_) content assay kit (ml016967), insect cytochrome P450 (CYP450) ELISA kit (ml603192), glutathione S-transferase (GST) assay kit (ml076443), carboxylesterase (CarE) activity assay kit (ml076534), peroxidase (POD) assay kit (ml076323), and catalase (CAT) assay kit (ml076329) were all purchased from Shanghai Enzyme-linked Biotechnology Co., Ltd. (Shanghai, China). The superoxide dismutase (SOD, total) assay kit (A001-1-2) was obtained from Nanjing Jiancheng Bioengineering Institute (Nanjing, China).

### 2.3. Experimental Instruments

This study utilized the following experimental apparatus: an SPX-250B-G microcomputer light incubator, a stereomicroscope (MZ101), a microscope camera (MSX2), an FB224 electronic balance (accurate to 1/10,000), and a drying oven.

### 2.4. Experimental Methods

#### 2.4.1. Determination of Feeding Preference of *S. litura* to TSN

The feeding preference of *S. litura* to TSN was evaluated using a two-choice mixed-diet assay. A mixed diet containing 25 μg/g of TSN was prepared, with the normal artificial diet serving as the control. Cylindrical diet blocks, 1 cm in diameter and 0.5 cm in height, were prepared using a sterile hole puncher. One untreated feed block was placed at the center of the left side of a 9 cm Petri dish, and one TSN-treated feed block was placed at the center of the right side. To avoid positional bias, the left–right positions of the treated and control diet blocks were alternated among replicates. For each replicate, 25 uniformly sized third-instar larvae of *S. litura*, starved for 4 h before the assay, were released at the center of the Petri dish. Each Petri dish was considered one independent biological replicate. The assay was performed with three independent biological replicates. The Petri dishes were sealed with plastic wrap and Parafilm, with several small holes made to allow ventilation, and then maintained in an incubator under standard rearing conditions. Photographs were taken every 12 h, and the numbers of larvae present on the TSN-treated and control diet blocks were recorded until the control feed block was completely consumed.

#### 2.4.2. Determination of the Effects of TSN on the Growth and Development of *S. litura*

The effects of TSN on the growth and development of *S*. *litura* larvae were evaluated using a diet incorporation bioassay. TSN was dissolved in dimethyl sulfoxide (DMSO) to prepare a 10 mg/mL stock solution. Appropriate volumes of the TSN stock solution were thoroughly mixed with the artificial diet to obtain final concentrations of 6.25, 12.5, 25, 50, and 100 μg/g diet [31]. The final concentration of DMSO was kept identical in the control and all TSN-treated diets at 0.005% (*v*/*w*). If Tween-80 was used as a dispersant, its final concentration was also kept identical in the control and all TSN-treated diets at 0.05% (*v*/*w*). In a preliminary assay, no significant difference was observed between larvae fed the untreated artificial diet and those fed the solvent-containing diet. Therefore, the solvent-containing diet was used as the control in the formal experiment.

Uniformly sized third-instar larvae of *S. litura* were starved for 4 h before treatment. The initial body weight of the larvae was recorded immediately before treatment using a precision analytical balance (0.1 mg accuracy), and the mean initial weight per replicate was calculated based on 10 randomly selected larvae. Each replicate consisted of 50 larvae and was treated as one independent biological replicate. Larvae were not individually tracked over time. Larvae were provided with fresh treated or control diet ad libitum and maintained under controlled environmental conditions.

Mortality was recorded daily, and dead larvae were removed immediately from the rearing containers and counted cumulatively for survival analysis. Final larval body weight was recorded at the last larval instar stage prior to pupation using the same sampling procedure. Different larval cohorts were used for each sampling time point to ensure the independence of observations.

The entire experiment was independently repeated three times. Based on the collected data, the pupation rate, pupal deformity rate, adult emergence rate, and adult deformity rate were calculated for each TSN concentration treatment. A positive control was not included in this study because the focus was on concentration-dependent responses of TSN; however, this limitation is acknowledged in the discussion.

#### 2.4.3. Determination of the Effects of TSN on Nutritional Indices of *S. litura*

The effect of TSN on the nutritional efficiency of *S. litura* was evaluated using the mixed-feed poisoning method. Treated diets containing 25, 50, and 100 μg/g TSN were prepared as described in Section 2.4.2, with the untreated diet serving as the control.

Fourth-instar larvae of similar size and developmental stage were selected and starved for 6 h before testing. Ten larvae were placed in each rearing box as one treatment, and each treatment was replicated five times. The fresh body weight of the larvae was recorded using an electronic balance. Cylindrical diet blocks of identical weight were punched out using a hole puncher for each treatment group and the control group and placed into the rearing containers. Feed was replaced every 24 h, and after 48 h of feeding, the feed was removed. Larvae were then starved for an additional 12 h before being flash-frozen in liquid nitrogen. The uneaten feed, feces, and frozen larvae were dried in an oven at 80 °C until a constant weight was reached. After starvation, the larvae were weighed to obtain their fresh weight. They were then dried and weighed again to determine their dry weight, which was used as the pre-treatment larval baseline dry weight. Separately, larvae were weighed before feeding (fresh weight), dried, and reweighed to obtain the pre-treatment larval dry weight. Additionally, three feed blocks were set aside, weighed fresh, dried, and reweighed to determine the pre-feeding feed dry weight.

Based on these data, the following nutritional indices for 10 larvae over 48 h were calculated: approximate digestibility (AD), efficiency of conversion of ingested food (ECI), efficiency of conversion of digested food (ECD), relative growth rate (RGR), and relative consumption rate (RCR).

#### 2.4.4. Changes in Enzyme Activity of *S. litura* After TSN Treatment at Different Time Intervals

The mixed-feed poisoning method was used to prepare a TSN-treated diet with a concentration of 50 μg/g, while the untreated artificial diet served as the control. Fourth-instar larvae of *S. litura* of uniform size were starved for 4 h before being fed either the treated or control diet. After 24 h, 48 h, and 72 h of treatment, 20 larvae from each group were dissected under sterile conditions on ice to collect their midguts. The dissected tissues were flash-frozen in liquid nitrogen for 10 min and then stored at −80 °C. The activities of three detoxification enzymes [32]—cytochrome P450 (spectrophotometric method), glutathione S-transferase (micro-method), and carboxylesterase (micro-method)—three digestive enzymes [33]—lipase (micro-method), trypsin (spectrophotometric method), and amylase (micro-method)—and three protective enzymes [34]—superoxide dismutase (spectrophotometric method), peroxidase (microplate method), and catalase (spectrophotometric method)—were determined according to the operating procedures of the respective assay kits. The above experiment was repeated three times. Full experimental details are available in the Supplementary Materials.

### 2.5. Data Handling

#### 2.5.1. Feeding Preference Analysis of *S. litura* Larvae Exposed to TSN

The feeding preference of *S. litura* larvae for normal and TSN-treated diets was evaluated using a dual-choice bioassay. The feeding preference index was calculated according to the following formula:

(1) $$\mathrm{Preference}\;\mathrm{Index}\left(\%\right)=\frac{\mathrm{Number}\;\mathrm{of}\;\mathrm{larvae}\;\mathrm{feeding}\;\mathrm{on}\;\mathrm{each}\;\mathrm{treatment}}{\mathrm{Total}\;\mathrm{number}\;\mathrm{of}\;\mathrm{larvae}\;\mathrm{per}\;\mathrm{treatment}}\times 100\%$$

#### 2.5.2. Calculation of Growth and Development Indices of *S. litura*

The growth and development parameters of *S*. *litura* larvae were calculated according to the following formulas.

(2) $$\mathrm{Pupation}\;\mathrm{percent}\left(\%\right)=\frac{\mathrm{Number}\;\mathrm{of}\;\mathrm{pupation}\;\mathrm{per}\;\mathrm{replicate}}{\mathrm{Total}\;\mathrm{number}\;\mathrm{of}\;\mathrm{test}\;\mathrm{insects}\;\mathrm{per}\;\mathrm{replicate}}\times 100\%$$

(3) $$\mathrm{Pupal}\;\mathrm{deformity}\;\mathrm{percent}\left(\%\right)=\frac{\mathrm{Number}\;\mathrm{of}\;\mathrm{deformed}\;\mathrm{pupae}\;\mathrm{per}\;\mathrm{repeat}}{\mathrm{Number}\;\mathrm{of}\;\mathrm{pupae}\;\mathrm{per}\;\mathrm{repeat}}\times 100\%$$

(4) $$\mathrm{Adult}\;\mathrm{emergence}\;\mathrm{percent}\left(\%\right)=\frac{\mathrm{Number}\;\mathrm{of}\;\mathrm{emerged}\;\mathrm{adults}\;\mathrm{per}\;\mathrm{repeat}}{\mathrm{Number}\;\mathrm{of}\;\mathrm{pupae}\;\mathrm{per}\;\mathrm{replicate}}\times 100\%$$

(5) $$\mathrm{Adult}\;\mathrm{deformity}\;\mathrm{percent}\left(\%\right)=\frac{\mathrm{Number}\;\mathrm{of}\;\mathrm{adults}\;\mathrm{per}\;\mathrm{duplication}\;\mathrm{deformity}}{\mathrm{Number}\;\mathrm{of}\;\mathrm{adult}\;\mathrm{eclosion}\;\mathrm{per}\;\mathrm{replicate}}\times 100\%$$

#### 2.5.3. Calculation for Nutritional Effect Indices of *S*. *litura*

The nutritional effect indices of *S. litura* larvae were calculated according to the method described by Waldbauer (1968), with slight modifications. The formulas were as follows:

(6) $$\mathrm{Approximate}\;\mathrm{Digestibility}\;\left(AD\right)\left(\%\right)=\frac{\left(I-F\right)}{I}\times 100\%$$

(7) $$\mathrm{Efficiency}\;\mathrm{of}\;\mathrm{Conversion}\;\mathrm{of}\;\mathrm{Ingested}\;\mathrm{Food}\left(\mathrm{ECI}\right)\left(\%\right)=\frac{G}{I}\times 100\%$$

(8) $$\mathrm{Efficiency}\;\mathrm{of}\;\mathrm{Conversion}\;\mathrm{of}\;\mathrm{Digested}\;\mathrm{Food}\left(\mathrm{ECD}\right)\left(\%\right)=\frac{G}{\left(I-F\right)}\times 100\%$$

(9) $$\mathrm{Relative}\;\mathrm{Growth}\;\mathrm{Rate}\left(\mathrm{RGR}\right)\left(\%\right)=\frac{G}{B\times T}\times 100\%$$

(10) $$\mathrm{Relative}\;\mathrm{Consumption}\;\mathrm{Rate}\left(\mathrm{RCR}\right)\left(\%\right)=\frac{I}{B\times T}\times 100\%$$

*G* represents the larval weight gain, calculated as *G* = dry weight of larvae after treatment − dry weight of larvae before treatment; *B* represents the mean larval dry weight during the experimental period, calculated as *B* = (dry weight before treatment + dry weight after treatment)/2; *I* represents the food intake, calculated as *I* = dry weight of diet before feeding − dry weight of diet after feeding; *F* represents the dry weight of feces; and *T* represents the feeding duration in days.

#### 2.5.4. Analysis and Graphing

Feeding preference data were analyzed using independent-samples Student’s *t*-test (SPSS 20.0, IBM Corp., Armonk, NY, USA), comparing the control and TSN-treated groups at each time point (12 h and 24 h). Proportional data (pupation, emergence, deformity rates) were analyzed using generalized linear models (GLMs) with a binomial distribution and logit link function. Continuous variables (weight, enzyme activity, and nutritional indices) were analyzed using one-way or two-way ANOVA, depending on experimental design. Post hoc comparisons were performed using Duncan’s test when the assumptions of homogeneity were met; otherwise, Tamhane’s T2 test was applied.

## 3. Results

### 3.1. Effect of TSN on the Feeding Preference of S. litura

**Figure 1:**
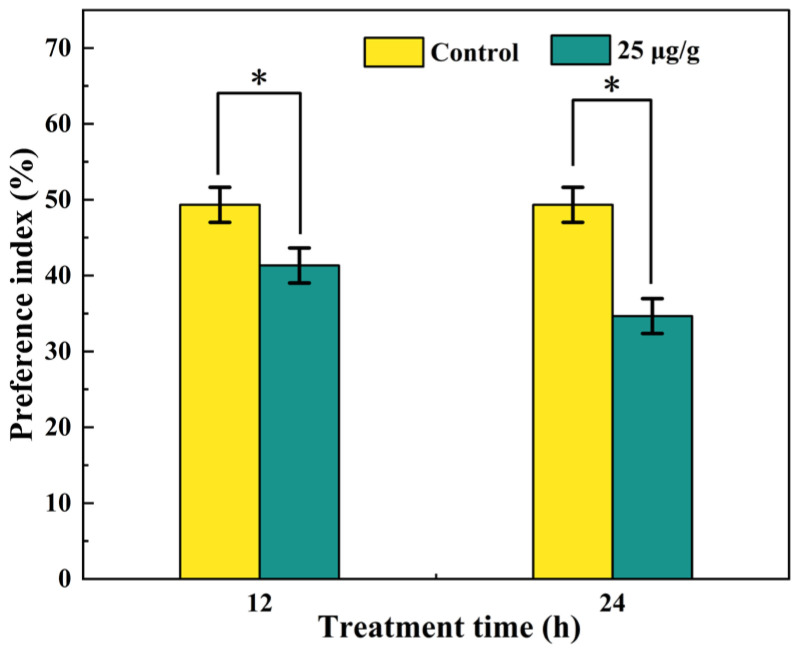
Feeding preference of *S. litura* larvae exposed to TSN (25 μg/g) for 12 h and 24 h. Data are presented as mean ± standard error (SE). Differences between treatments at each time point were analyzed using independent Student’s *t*-test (*p* < 0.05); values marked with * indicate significant differences at *p* < 0.05.

The feeding preference index of *S. litura* remained unchanged in the control group at 49.3% after both 12 and 24 h. In contrast, larvae exposed to TSN exhibited a dual behavioral response, including both repellence and feeding inhibition. The feeding preference index of *S. litura* significantly decreased in the 25 μg/g TSN treatment group from 41.3% at 12 h to 34.7% at 24 h, representing a reduction of 6.6% (Figure 1, Table S1). This decline indicates a gradual shift in feeding away from the TSN-supplemented feed, suggesting that TSN exerts a repellent (avoidance) effect. Moreover, feeding inhibition was observed based on consumption dynamics. The normal feed was completely consumed by 36 h in the treatment group. In contrast, the feed still remained in the control group at 36 h and was completely consumed by 48 h. However, the TSN-supplemented feed was still not fully consumed at 48 h.

In summary, these results indicate that *S. litura* larvae prefer the normal feed to the TSN-supplemented feed, suggesting that TSN induces both repellence and feeding inhibition rather than attraction.

### 3.2. Effects of TSN on the Growth and Development of S. litura Larvae

#### 3.2.1. Growth-Inhibitory Effect of TSN on *S. litura* Larvae

**Figure 2:**
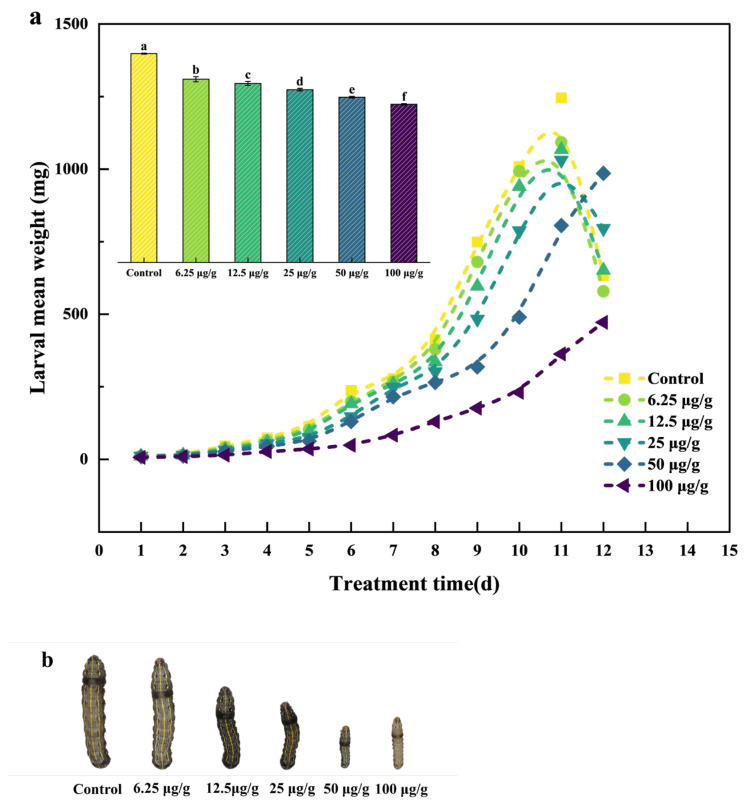
Effects of TSN on the growth of *S. litura* larvae: (**a**) Dynamic changes in larval mean body weight (mg) over a 12-day exposure period under different TSN concentrations (control, 6.25, 12.5, 25, 50, and 100 μg/g). Each point represents the mean larval weight calculated from 10 randomly selected individuals per replicate. Different letters indicate significant differences (*p* < 0.05) in the maximum total weight of 10 larvae between the TSN-treated and control groups, as determined by one-way ANOVA. (**b**) Morphology of larvae on day 7 after treatment—left: control group; right: TSN-treated group.

After treatment with feeds containing 6.25, 12.5, 25, 50, and 100 μg/g TSN, the body weight of *S. litura* larvae was significantly lower than that of the control group (*p* < 0.05, Figure 2a and Table S2-I). The larval weight increased continuously during the early feeding period in all groups. However, the growth trajectories of the TSN-treated larvae gradually diverged from that in the control group, particularly after day 6, indicating a concentration-dependent inhibition of larval growth. On day 7 after treatment, the larvae in the control group had reached the late sixth instar stage, whereas those in the TSN-treated group were still in the fifth to sixth instar stage (Figure 2a,b). This developmental delay suggested that TSN not only reduced larval biomass accumulation but also slowed larval developmental progression.

The maximum weight of larvae in the control group was 1245.4 mg per larva. In contrast, the maximum mean larval weights in the 6.25, 12.5, 25, 50, and 100 μg/g TSN-treated group were 1092.7, 1067.2, 1030.6, 985.6, and 472.1 mg per larva, respectively. These values represented 0.88-, 0.86-, 0.83-, 0.79-, and 0.38-fold of the control level, respectively (Figure 2a and Table S2-II). The strongest inhibitory effect was observed at 100 μg/g TSN, where larval body weight was reduced by approximately 62% compared with that in the control group. These results indicate that TSN treatment significantly inhibits the growth of *S. litura* larvae.

#### 3.2.2. Effects of TSN on Pupal Duration and Pupal Weight of *S. litura* Larvae

**Figure 3:**
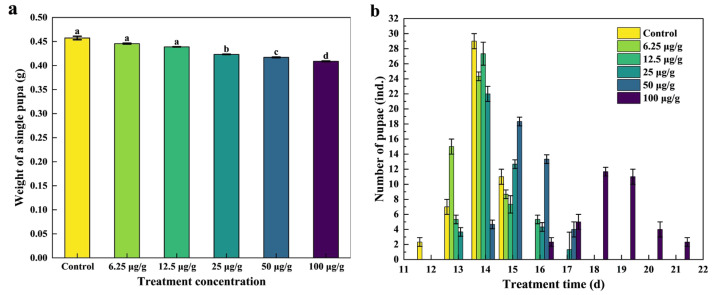
Effects of TSN on pupation timing and pupal weight of *Spodoptera litura*: (**a**) Different letters indicate significant differences (*p* < 0.05) in the weight of a single pupa between TSN-treated and control groups, as determined by one-way ANOVA; (**b**) Comparison of pupation duration and the number of pupae between the TSN-treated and control larvae.

In the control group, *S. litura* larvae began pupation on day 12. The number of pupating larvae first increased and then decreased on a daily basis, reaching a peak of approximately 29 pupae on day 14. Different concentrations of TSN impacted the timing of pupation: larvae treated with 6.25, 12.5, and 25 μg/g TSN pupated 2 days later than that in the control group (beginning on day 13); larvae treated with 50 μg/g TSN pupated 2 days later (on day 14); and those treated with 100 μg/g TSN pupated 4 days later (on day 16). The pupation pattern in the TSN-treated group was similar to that in the control group, showing an initial increase followed by a subsequent decrease in the number of pupae. However, the peak number of pupae decreased, and the time of peak pupation was delayed as follows: 27 pupae on day 14 at 6.25 μg/g TSN, 24 on day 14 at 12.5 μg/g TSN, 22 on day 14 at 25 μg/g TSN, 18 on day 15 at 50 μg/g TSN, and 12 on day 18 at 100 μg/g TSN (Figure 3a, Table S3-II).

The average pupal weights in the TSN-treated group was 0.44559, 0.43889, 0.42323, 0.41704, and 0.40881 g, respectively (Figure 3b and Table S3-I), whereas that in the control group was 0.45734 g, 1.03, 1.04, 1.08, 1.09, and 1.12 times higher than those in the treatment groups (*p* < 0.05).

#### 3.2.3. Effects of TSN on Pupation, Pupal Malformation, Eclosion, and Adult Malformation Rates in *S. litura*

**Figure 4:**
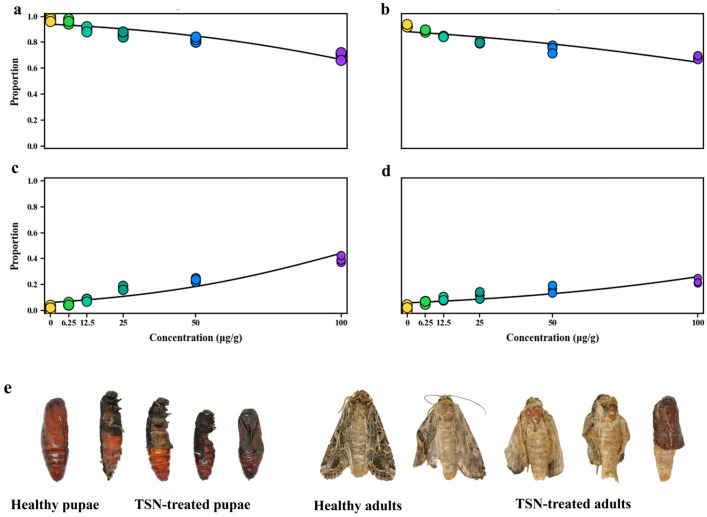
Dose-dependent impacts of TSN on the developmental performance of *S. litura*. (**a**) Pupation, (**b**) adult emergence, (**c**) pupal deformity, and (**d**) adult deformity across a gradient of TSN concentrations (0–100 μg/g). Points represent observed proportions from independent replicates. Solid curves represent fitted values from generalized linear models (GLMs) assuming a binomial distribution with a logit link function. All models revealed significant dose-dependent effects (*p* < 0.001), indicating reduced pupation and emergence and increased developmental deformities with increasing TSN concentration. (**e**) Normal and malformed pupae and adults of *S. litura*.

The developmental responses of *S. litura* exhibited a clear concentration-dependent pattern following TSN treatment, as demonstrated by generalized linear model (GLM) analyses with a binomial distribution (Figure 4a–d).

Binomial GLM analysis confirmed a strong negative correlation between TSN concentration and pupation probability [*β* = −0.0205, odds ratio (OR) = 0.9797, *p* < 0.001]. This indicated that each unit increase in TSN concentration resulted in a consistent reduction in the likelihood of successful pupation. A similar declining trend was observed for adult emergence. The GLM results showed a significant negative correlation between TSN concentration and emergence rate (*β* = −0.0141, OR = 0.9860, *p* < 0.001), indicating that higher TSN exposure reduced the probability of successful eclosion. Although the effect size was slightly lower than that observed for pupation, the overall trend remained consistent and statistically robust (Table S4).

In contrast, the probability of developmental abnormalities increased significantly with increasing TSN concentration. The pupal deformity rate exhibited a strong positive dose–response relationship (*β* = 0.0249, OR = 1.0252, *p* < 0.001), indicating that each unit increase in concentration substantially elevated the probability of malformed pupae. Similarly, the adult deformity rate also increased significantly with dose (*β* = 0.0173, OR = 1.0174, *p* < 0.001), demonstrating a consistent increase in abnormal adult emergence at higher TSN concentrations (Table S4).

These results indicate that TSN reduces pupation and eclosion rates while increasing the pupal and adult malformation rates in *S. litura*, with stronger effects observed at higher concentrations. Under TSN treatment, pupae exhibited abnormal molting, incomplete ecdysis, and shriveled bodies, whereas adults displayed wing deformities, failure to fully expand their wings, and had difficulty shedding the pupal cuticle (Figure 4e).

### 3.3. Effects of TSN on Nutritional Indices of S. litura Larvae

The nutritional indices of fourth-instar larvae of *S. litura* after 48 h of feeding on artificial diets containing different concentrations of TSN are shown in Table 1. The TSN-treated larvae exhibited significant changes in all measured nutritional indices compared with those in the control group (*p* < 0.05). The efficiency of conversion of digested food, relative growth rate (RGR), and relative consumption rate (RCR) were significantly reduced in all TSN-treated groups (*p* < 0.05), displaying a clear concentration-dependent decreasing trend with increasing TSN concentrations. In contrast, approximate digestibility (AD) was significantly higher in all TSN-treated groups compared with the control group (*p* < 0.05), exhibiting a progressive increase with increasing TSN concentrations.

A nonlinear response pattern was observed for the efficiency of conversion of ingested food (ECI). Despite an increase in ECI in larvae treated with 25 and 50 μg/g TSN compared with the control group, a slight decrease was observed in larvae treated with 100 μg/g TSN. Nevertheless, ECI remained significantly different in all TSN-treated groups compared with the control group (*p* < 0.05) (Table 1).

### 3.4. Effects of TSN on Enzyme Activities of S. litura

#### 3.4.1. Changes in Digestive Enzymes of *S. litura* Treated with TSN for Different Time Periods

The effects of TSN on the activities of three digestive enzymes—lipase (LPS), trypsin, and α-amylase—in the midgut of fourth-instar *S. litura* larvae were examined at 24, 48, and 72 h post-treatment using two-way analysis of variance (ANOVA; treatment × time). Detailed statistical results are presented in Tables S5-II–S5-X. The overall ANOVA results for each enzyme are summarized in Table S5.

**Figure 5:**
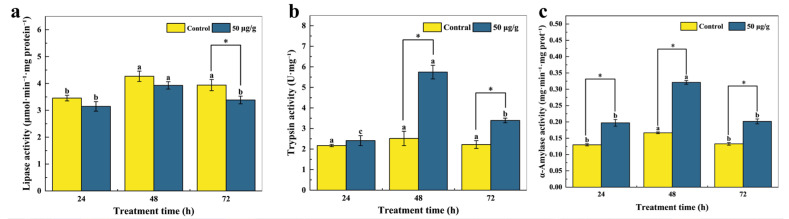
Changes in digestive enzyme activities of *S. litura* after treatment with TSN at different time points. (**a**) Lipase activity, (**b**) trypsin activity, and (**c**) α-amylase activity in the control and 50 μg/g treatment groups at 24, 48, and 72 h. Error bars indicate the standard error (SE). Different lowercase letters indicate significant differences among treatment times (24, 48, and 72 h) under the same concentration (*p* < 0.05). Asterisks (*) indicate significant differences between the control and 50 μg/g treatment groups at the same time point (*p* < 0.05).

Two-way ANOVA revealed significant main effects of treatment [*F*(1, 12) = 26.493, *p* < 0.001, *η*^2^_p_ = 0.688] and time [*F*(2, 12) = 35.133, *p* < 0.001, *η*^2^_p_ = 0.854], but no significant treatment × time interaction [*F*(2, 12) = 0.992, *p* = 0.399, *η*^2^_p_ = 0.142] effect on lipase activity. As shown in Figure 5a, the temporal trend in lipase activity was similar in both the control and TSN-treated groups, with a significant increase at 48 h followed by a decrease at 72 h. However, lipase activity in the TSN-treated group was consistently lower than that in the control group at all three time points, reaching a statistically significant difference at 72 h. These results indicate an overall suppressive effect of TSN treatment on lipase activity, particularly during the later phase of exposure.

In contrast, trypsin activity exhibited a strong interaction between treatment and time [*F*(2, 12) = 61.386, *p* < 0.001, *η*^2^_p_ = 0.911], indicating that the effect of TSN on trypsin activity was highly time-dependent. As shown in Figure 5b, no significant change in trypsin activity was observed in the control group over time. However, trypsin activity increased dramatically from 2.41 U·mg^−1^ at 24 h to 5.75 U·mg^−1^ at 48 h and then decreased to 3.39 U·mg^−1^ at 72 h in the TSN-treated group. TSN-treated larvae displayed significantly higher trypsin activity at 48 h than the control group (S5-III). These findings suggest that TSN exposure induced a strong but transient upregulation of trypsin activity, peaking at 48 h and declining thereafter, though it still remained above the control level at 72 h.

A similar pattern of significant interaction was observed for α-amylase activity [*F*(2, 12) = 94.473, *p* < 0.001, *η*^2^_p_ = 0.940]. As depicted in Figure 5c, both the control and TSN-treated groups exhibited a significant increase in α-amylase activity at 48 h, followed by a decrease at 72 h. However, the magnitude of change was considerably greater in the TSN-treated group than in the control group. At every time point examined, the α-amylase activity in the TSN-treated group was significantly higher than that in the control group. These results indicate that TSN treatment elicited a sustained and pronounced stimulation of α-amylase activity throughout the 72 h experimental period.

Overall, the three digestive enzymes exhibited differential responses to TSN exposure: lipase activity was moderately suppressed, whereas trypsin and α-amylase activities were significantly induced, with the latter two displaying clear time-dependent patterns and strong treatment–time interactions. These results suggest that TSN disrupted normal digestion in *S. litura* larvae, potentially by differentially modulating the activities of key hydrolytic enzymes in the midgut.

#### 3.4.2. Effects of TSN on Detoxification Enzymes of *S. litura*

The activities of three representative detoxification enzymes—CYP450, glutathione S-transferase (GST), and carboxylesterase (CarE)—in the midgut of fourth-instar *S. litura* larvae were measured at 24, 48, and 72 h after treatment with 50 μg/g TSN. Two-way ANOVA (treatment × time) was performed for each enzyme. Detailed statistical results are presented in Tables S5-II–S5-X. The overall ANOVA results for each enzyme are summarized in Table S5.

**Figure 6:**
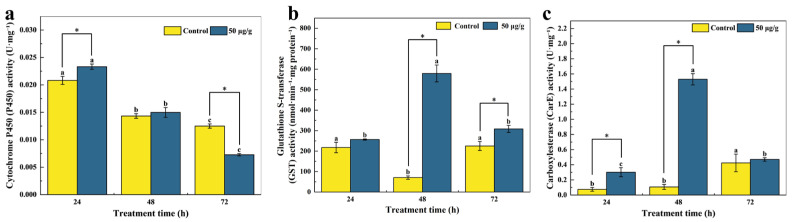
Changes in detoxification enzyme activities of *S. litura* after treatment with TSN at different time points. (**a**) Cytochrome P450 (P450), (**b**) glutathione S-transferase (GST), and (**c**) carboxylesterase (CarE) activities in the control and 50 μg/g treatment groups at 24, 48, and 72 h. Error bars indicate the standard error (SE). Different lowercase letters indicate significant differences among treatment times (24, 48, and 72 h) under the same concentration (*p* < 0.05). Asterisks (*) indicate significant differences between the control and 50 μg/g treatment groups at the same time point (*p* < 0.05).

Two-way ANOVA revealed a nonsignificant main effect of treatment [*F*(1, 12) = 3.273, *p* = 0.096, *η*^2^_p_ = 0.214], a highly significant main effect of time [*F*(2, 12) = 359.273, *p* < 0.001, *η*^2^_p_ = 0.984], and a significant treatment × time interaction effect [*F*(2, 12) = 42.545, *p* < 0.001, *η*^2^_p_ = 0.876] on CYP450 activity. As shown in Figure 6a, CYP450 activity in both the control and TSN-treated groups decreased progressively over time. In the control group, the activity declined from 0.021 U·mg^−1^ at 24 h to 0.013 U·mg^−1^ at 72 h. In the TSN-treated group, a more pronounced decrease was observed, from 0.023 U·mg^−1^ at 24 h to 0.007 U·mg^−1^ at 72 h. Between-group comparisons revealed that CYP450 activity in the treatment groups was significantly higher than that in the control group at 24 h, not significantly different at 48 h, but significantly lower at 72 h. These results indicate that the effect of TSN on CYP450 activity is time-dependent: an early transient elevation is followed by marked suppression at later exposure times, consistent with the significant interaction term.

Two-way ANOVA indicated significant main effects of treatment [*F*(1, 12) = 373.582, *p* < 0.001, *η*^2^_p_ = 0.969] and time [*F*(2, 12) = 22.474, *p* < 0.001, *η*^2^_p_ = 0.789], and a highly significant interaction effect [*F*(2, 12) = 189.464, *p* < 0.001, *η*^2^_p_ = 0.969] on GST activity. As depicted in Figure 6b, GST activity in the control group decreased markedly at 48 h [to 70.763 nmol·min^−1^·mg·protein^−1^] compared with that at 24 and 72 h. In contrast, GST activity in the TSN-treated group increased sharply at 48 h, reaching 579.677 nmol·min^−1^·mg·protein^−1^, and then declined at 72 h. The treatment groups exhibited significantly higher GST activity than the control groups at all three time points, with statistically significant differences at 48 h and 72 h. These findings demonstrate that TSN exposure strongly induced GST activity, particularly at the 48 h time point, indicating an enhanced detoxification response.

Two-way ANOVA revealed significant main effects of treatment [*F*(1, 12) = 347.312, *p* < 0.001, *η*^2^_p_ = 0.967] and time [*F*(2, 12) = 145.410, *p* < 0.001, *η*^2^_p_ = 0.960], and a significant interaction effect [*F*(2, 12) = 203.146, *p* < 0.001, *η*^2^_p_ = 0.971] on CarE activity. As shown in Figure 6c, CarE activity in the control group remained relatively stable at 24 and 48 h (0.075 and 0.107 U·mg^−1^, respectively) but increased substantially at 72 h (0.424 U·mg^−1^). In the TSN-treated group, CarE activity exhibited a dramatic surge at 48 h (1.529 U·mg^−1^), followed by a decrease at 72 h (0.470 U·mg^−1^), with significant differences across time points. Compared with the control group, the TSN-treated group displayed significantly higher CarE activity at 24 h and 48 h, whereas no significant difference was observed at 72 h. This pattern indicates that TSN treatment caused a strong but transient induction of CarE activity, peaking at 48 h and returning to near-control levels by 72 h.

Collectively, these results demonstrate that TSN exposure differentially modulated the activities of the three detoxification enzymes in *S. litura* larvae. Although CYP450 activity was significantly suppressed at 72 h, both GST and CarE activities were significantly induced, especially at the 48 h time point. The distinct response patterns suggest that TSN might impair the CYP450-mediated detoxification pathway while simultaneously activating GST- and CarE-dependent protective mechanisms, reflecting a complex physiological adaptation of the insect to TSN stress.

#### 3.4.3. Effects of TSN on the Activities of Protective Enzymes of *S. litura* Treated with TSN for Different Times

The activities of three major antioxidant/protective enzymes—superoxide dismutase (SOD), peroxidase (POD), and catalase (CAT)—in the midgut of fourth-instar *S. litura* larvae were determined at 24, 48, and 72 h after exposure to 50 μg/g TSN. Two-way ANOVA (treatment × time) was performed for each enzyme. Detailed statistical results are presented in Tables S5-II–S5-X. The overall ANOVA results for each enzyme are summarized in Table S5.

**Figure 7:**
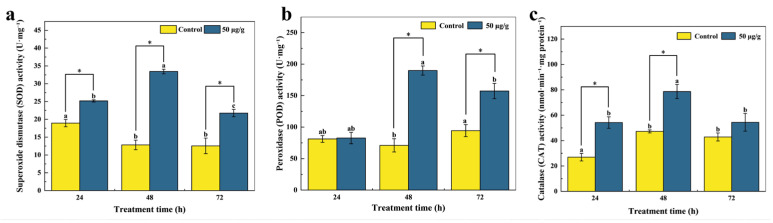
Changes in protective enzyme activities of *S. litura* following TSN treatment at different time intervals. (**a**) Superoxide dismutase (SOD), (**b**) peroxidase (POD), and (**c**) catalase (CAT) activities in the control and 50 μg/g treatment groups at 24, 48, and 72 h. Error bars indicate the standard error (SE). Different lowercase letters indicate significant differences among treatment times (24, 48, and 72 h) under the same concentration (*p* < 0.05). Asterisks (*) indicate significant differences between the control and 50 μg/g treatment groups at the same time point (*p* < 0.05).

Two-way ANOVA revealed significant main effects of treatment [*F*(1, 12) = 427.780, *p* < 0.001, *η*^2^_p_ = 0.973] and time [*F*(2, 12) = 40.073, *p* < 0.001, *η*^2^_p_ = 0.870], as well as a significant treatment × time interaction effect [*F*(2, 12) = 56.782, *p* < 0.001, *η*^2^_p_ = 0.904], on SOD activity. As shown in Figure 7a, SOD activity in the control group decreased from 18.943 U·mg^−1^ at 24 h to 12.838 U·mg^−1^ at 48 h, with no significant further change at 72 h. In the TSN-treated group, SOD activity increased significantly from 25.181 U·mg^−1^ at 24 h to a peak of 33.443 U·mg^−1^ at 48 h, followed by a sharp decline to 21.768 U·mg^−1^ at 72 h. At every time point examined, SOD activity in the TSN-treated group was significantly higher than that in the control group. These results indicate that TSN exposure consistently induces SOD activity, with the most pronounced stimulation occurring at 48 h.

Two-way ANOVA demonstrated significant main effects of treatment [*F*(1, 12) = 197.637, *p* < 0.001, *η*^2^_p_ = 0.943] and time [*F*(2, 12) = 50.680, *p* < 0.001, *η*^2^_p_ = 0.894], as well as a significant interaction effect [*F*(2, 12) = 60.834, *p* < 0.001, *η*^2^_p_ = 0.910] on POD activity. As depicted in Figure 7b, POD activity in the control group remained relatively stable across the three time points (ranging from 70.999 to 94.405 U·mg^−1^), with only modest variation. In contrast, POD activity in the TSN-treated group increased dramatically from 82.660 U·mg^−1^ at 24 h to a peak of 189.871 U·mg^−1^ at 48 h, and then declined to 157.322 U·mg^−1^ at 72 h. Between-group comparisons showed that POD activity was significantly higher in the treated groups than in the control group at both 48 h and 72 h, whereas no significant difference was observed at 24 h. These findings indicate that TSN treatment elicited a strong and sustained induction of POD activity, with the peak response at 48 h.

Two-way ANOVA revealed significant main effects of treatment [*F*(1, 12) = 123.178, *p* < 0.001, *η*^2^_p_ = 0.911] and time [*F*(2, 12) = 38.462, *p* < 0.001, *η*^2^_p_ = 0.865], as well as a significant interaction effect [*F*(2, 12) = 8.237, *p* = 0.006, *η*^2^_p_ = 0.579], on CAT activity. As shown in Figure 7c, CAT activity in the control group increased from 26.957 nmol·min^−1^·mg·protein^−1^ at 24 h to 47.244 nmol·min^−1^·mg·protein^−1^ at 48 h and then slightly decreased to 42.850 nmol·min^−1^·mg·protein^−1^ at 72 h. In the TSN-treated group, CAT activity rose from 54.220 nmol·min^−1^·mg·protein^−1^ at 24 h to a maximum of 78.626 nmol·min^−1^·mg·protein^−1^ at 48 h, followed by a significant reduction to 54.380 U/mg at 72 h. The TSN-treated group exhibited significantly higher CAT activity than the control group at both 24 h and 48 h, whereas the difference at 72 h was not statistically significant. These results indicate that TSN treatment induced a strong early increase in CAT activity (at 24 and 48 h), which subsequently declined to control levels by 72 h.

Collectively, the three antioxidant enzymes exhibited distinct but overlapping response patterns to TSN treatment. SOD and POD activities were significantly elevated at all time points examined (with the exception of POD activity at 24 h), whereas CAT induction was more transient, returning to control levels by 72 h. All three enzymes reached their highest activities at 48 h post-treatment, suggesting that this time point represented the peak of the antioxidant defense response. These results demonstrate that TSN exposure imposed significant oxidative stress on *S. litura* larvae, triggering a coordinated upregulation of protective enzyme systems to counteract the associated cellular damage.

## 4. Discussion

Plant-derived pesticides are bioproducts formulated from active compounds extracted from plants [35]. They are safe for nontarget organisms, have minimal residues, and are less likely to induce pest resistance, making them increasingly important research targets [36]. Thus, exploring the application of plant-derived pesticides in managing *S. litura* is highly significant.

Studies have reported the antifeedant activity of TSN against agricultural pests. For example, significant antifeedant activity has been documented in *Pieris rapae* [37], *S. frugiperda* [21,38], *C. formicarius* [39], and other lepidopteran species [40]. TSN can damage the intestinal epithelium of insects and inhibit the activities of esterases, CYP450 enzymes, and proteases, leading to digestive and metabolic disorders [41]. Shi Yuliang et al. used electrophysiological techniques to reveal the antifeedant mechanism of TSN: it acts on chemoreceptors associated with feeding in insects, reducing their sensitivity to food stimuli and thereby inhibiting their feeding behavior [42,43]. The results of this study are consistent with previous findings, indicating that TSN exhibited significant repellence toward n *S. litura*. After 24 h of exposure to a feed treated with TSN at a concentration of 25 μg/mL, the feeding preference index of *S. litura* larvae was only 34.7%. The normal (untreated) feed in the treatment group was almost completely consumed after 36 h, whereas the control diet was completely consumed after 48 h. In contrast, the TSN-treated diet remained partially unconsumed even after 48 h. However, the insecticidal activity of TSN against *S. litura* has not yet been reported.

In addition to its effect on feeding preference, TSN also exhibits direct toxicity to the growth and development of *S*. *litura* larvae. TSN has been shown to significantly inhibit larval growth and development in other pests. For instance, spraying 300 μg/mL TSN on fourth-instar *P. rapae* larvae for 48 h resulted in a pupation rate of only 15.38% after 5 days, with pupal weight reduced to 78.52% of that in the control group; fifth-instar larvae exhibited a pupation rate of 38.00%, and their pupal weight was 75.42% of that in the control group [44]. TSN at effective concentrations significantly suppressed the growth and development of Musca domestica larvae, causing developmental delay, an extended life cycle, reduced body size, delayed or failed pupation, pupal malformations, reduced eclosion rates, and adult malformations [7,45]. In this study, *S. litura* larvae were fed artificial feeds containing five different TSN concentrations (6.25, 12.5, 25, 50, and 100 μg/g) using the diet incorporation method. The inhibitory effect of TSN on larval growth was dose-dependent: low concentrations led to weak inhibition, whereas high concentrations strongly suppressed normal development. Larvae treated with 100 μg/g TSN had pupation delayed by 4 days, a maximum weight of 9.43 g for 10 larvae (76% of that in the control group), and a mean pupal weight reduced by 48.53 mg compared with that in the control group. High concentrations of 25–100 μg/g also significantly decreased pupation and eclosion rates and increased pupal and adult malformation rates.

In the present study, fourth-instar *S. litura* larvae treated with TSN showed significantly reduced food conversion efficiency, RGR, and RCR. This was likely because the antifeedant effects decreased food intake and impaired normal growth. Larval ECI was significantly higher than that in the control group but did not increase consistently with concentration; at 100 μg/g, ECI declined slightly. This pattern revealed a two-phase physiological response. The elevated ECI under moderate stress was consistent with the distinct effects of different compounds on the nutritional indices of insects. The impact of secondary metabolites, such as capsaicin and flavonoids, on the nutritional indices of *H. armigera* included a decrease in RCR and AD, an increase in ECI, and essentially no change in RGR [46]. However, this enhanced conversion efficiency did not translate into an increased RGR, indicating that the salvaged energy was not allocated to biomass gain. Instead, this “missing energy” was most likely diverted to meet the high metabolic cost of detoxification and cellular repair, representing a classic growth–defense trade-off. In infected locusts exposed to low temperatures, the combined demands of immune defense and the metabolic constraints associated with cold stress are hypothesized to lead to a rebalancing of nutritional indices. Specifically, AD increases to compensate for elevated energy demands, whereas ECI decreases as resources are diverted away from growth toward immune maintenance [47,48,49]. At the highest TSN concentration, the decline in ECI suggested that the compensatory mechanism collapsed, probably due to structural damage to the midgut epithelium and inhibition of digestive enzymes, beyond which physiological regulation is overridden by toxicity. The high efficiency might reflect compensatory utilization due to reduced consumption, whereas in controls, adequate intake allowed lower utilization. At very high concentrations, toxic effects likely reduced utilization capacity.

In this study, SOD, POD, and CAT activities significantly increased at 48 h after TSN treatment, indicating a strong early response, with a slight decline by 72 h possibly reflecting adaptation. Plant secondary metabolites can induce the expression or activity of detoxification enzymes in insects, thereby enhancing their adaptive capacity [50]. Toxic exposure can also induce protective enzyme responses in insects. Similar dynamics have been observed in other insect–pathogen or insect–pesticide interactions, including changes in SOD, POD, and CAT activities in *P. rapae* [51]; the activation–inhibition trend of antioxidant enzymes in *Holotrichia parallela* larvae exposed to EPN-Bt [52]; and changes in SOD, CAT, and POD activities in *P. rapae* under ethanol extract stress [53]. The initial surge in antioxidant enzymes is a direct consequence of the TSN-induced reactive oxygen species burst, whereas the subsequent decline likely reflects enzyme exhaustion or oxidative damage rather than simple adaptation, a temporal profile that mirrors the intensity and duration of the oxidative challenge imposed by various phytochemicals. Digestive enzyme activities reflect the ability of insects to process ingested nutrients. In this study, treatment with 50 μg/mL TSN did not significantly affect lipase activity but initially increased trypsin and α-amylase activities, followed by a decrease. This suggests that short-term stress from toosendanin initially enhances enzyme activity, but by 72 h, stronger toxic effects damage enzyme structure and reduce activity. Indeed, glutathione S-transferase (GST) and carboxylesterase (CarE) activities were higher in treated larvae than in the controls, consistent with this effect, whereas cytochrome P450 activity was significantly reduced, suggesting structural damage that impairs its detoxification function. This inverse correlation between Phase I (CYP450) and Phase II (GST and CarE) enzymes is particularly informative. The pronounced inhibition of CYP450 suggests that this enzyme complex may be a specific target of TSN, either through direct binding or structural disruption, thereby blocking a major oxidative detoxification pathway. In response, the insect appears to activate alternative detoxification routes—GST-mediated conjugation and CarE-mediated hydrolysis—as a compensatory redundancy strategy. Such pathway shifting is rarely observed with conventional neurotoxic insecticides, which typically induce CYP450s, highlighting a distinctive mode of action for this botanical compound. The temporary stimulation followed by the subsequent decline in digestive enzyme activity can be explained by an energetic trade-off: under acute stress, resources are transiently mobilized to digestion to counter the antifeedant effects, but as toxicity progresses and cellular damage accumulates, energy is increasingly redirected toward detoxification and immune maintenance, ultimately impairing digestive function.

A limitation of this study is the absence of a positive control. However, this does not affect the validity of the results, as the primary objective of this work was to evaluate the concentration-dependent physiological and biochemical responses of *S*. *litura* to TSN, rather than to compare its efficacy with that of synthetic insecticides. The use of multiple endpoints, including developmental parameters, digestive enzyme activities, detoxification enzymes, and antioxidant responses, provides a comprehensive assessment of TSN toxicity under controlled laboratory conditions.

## 5. Conclusions

In conclusion, this study showed that TSN significantly suppressed the feeding, growth, development, pupation, and adult emergence of *S. litura*. TSN-treated larvae exhibited reduced feeding preference, lower larval weight, delayed pupation, decreased pupation and emergence rates, and increased pupal and adult malformation rates, indicating a clear concentration-dependent inhibitory effect of TSN on development. Moreover, TSN altered nutritional indices by reducing food consumption, food conversion efficiency, and RGR, suggesting that its growth-inhibitory effect was closely related to impaired nutrient intake and utilization. TSN also impacted the activities of digestive, detoxification, and protective enzymes. The induction of several detoxification and antioxidant enzymes indicated that *S. litura* larvae could initiate physiological defense responses under TSN stress, whereas the reduction in CYP450 activity and the persistent inhibition of growth suggested that these responses could not completely offset the adverse impacts of TSN. These results indicate that TSN inhibits *S. litura* through multiple physiological pathways, including antifeedant activity, disruption of nutrient metabolism, interference with enzyme activity, and impairment of normal development. Therefore, TSN may serve as an environmentally friendly botanical insecticide for managing *S. litura*. Further field-based studies are required to confirm the practical efficacy, application strategy, and ecological safety of TSN.

## Figures and Tables

**Table 1:** Nutritional parameters of fourth-instar *S. litura* larvae after 48 h of feeding on artificial diets containing different concentrations of TSN.

TSN Concentration (μg/g)	AD (%)	ECD (%)	ECI (%)	RGR (mg/mg·d)	RCR (mg/mg·d)
Control	28.73532 ± 0.53116 c	79.77658 ± 2.64188 a	22.88181 ± 0.52235 b	0.57252 ± 0.00346 a	2.560695 ± 0.05494 a
25	39.36151 ± 1.03654 b	68.91138 ± 2.26361 b	27.03559 ± 0.30080 a	0.54849 ± 0.00619 b	2.02905 ± 0.01765 b
50	40.45137 ± 0.96692 b	67.52558 ± 1.39883 b	27.31050 ± 0.84705 a	0.53192 ± 0.00773 b	1.95177 ± 0.03566 b
100	45.74446 ± 0.79971 a	58.95567 ± 1.01953 c	26.94713 ± 0.38301 a	0.46647 ± 0.01046 c	1.73305 ± 0.05123 c

Notes: AD, approximate digestibility; ECD, efficiency of conversion of digested food; ECI, efficiency of conversion of ingested food; RCR, relative consumption rate; RGR, relative growth rate. Data are presented as the mean ± standard error. One-way ANOVA was used for statistical analysis, and multiple comparisons were performed using the Duncan’s multiple range test (DMRT). Within each column, different lowercase letters indicate significant differences (*p* < 0.05), while the same letters indicate no significant difference.

## Data Availability

The data presented in this study are available on request from the corresponding author.

## Supplementary Materials

The following supporting information can be downloaded at https://www.mdpi.com/article/10.3390/insects17070732/s1, Table S1: Effect of toosendanin on the feeding preference of *Spodoptera litura* larva; Table S2-I: Effect of toosendanin on maximum larval weight (g) and instar stage of *S. litura* on day 12 post treatment; Table S2-II Effects of different concentrations of toosendanin on the maximum larval selected individuals per replicate (mg) of *S. litura*; Table S3-I Effect of toosendanin on individual pupal weight per replicate (g) of *S. litura*; Table S3-II Effect of toosendanin at different concentrations on the number of pupated individuals of *S. litura*; Table S4 GLM analysis of developmental responses of *S. litura* exposed to TSN; Table S5-I. Effects of TSN on enzyme activities of *S. litura* larvae based on two-way ANOVA (Treatment × Time); Table S5-II Descriptive statistics (mean ± SD) of lipase activity in *S. litura* under different treatments and exposure times; Table S5-III Descriptive statistics (mean ± SD) of trypsin activity in *S. litura* under different treatments and exposure times; Table S5-IV Descriptive statistics (mean ± SD) of α-Amylase activity in *S. litura* under different treatments and exposure times; Table S5-V Detoxification statistics (mean ± SD) of CYP450 activity in *S. litura* under different treatments and exposure times; Table S5-VI Detoxification statistics (mean ± SD) of Glutathione S-transferase activity in *S. litura* under different treatments and exposure times. Table S5-VII Detoxification statistics (mean ± SD) of CarE in *S. litura* under different treatments and exposure times. Table S5-VIII Oxidative stress enzyme statistics (mean ± SD) of Superoxide dismutase (SOD) activity in *S. litura* under different treatments and exposure times; Table S5-IX Oxidative stress enzyme statistics (mean ± SD) of Peroxidase activity in *S. litura* under different treatments and exposure times; Table S5-X Oxidative stress enzyme statistics (mean ± SD) of Catalase (CAT) activity in *S. litura* under different treatments and exposure times.

## References

[1] Ghosh P. (2016). Virulence, UV Protection, Cross Infectivity and Shelf Life Studies of *SJNPV*. Master’s Thesis.

[2] Zhang M., Ren X., Hu H., Wang D., Song X., Ma Y., Ma X. (2023). Evaluation of cotton, sweet potato, peanut, and black nightshade on the fitness of *Spodoptera litura* Fabricius (Lepidoptera: Noctuidae). Int. J. Trop. Insect Sci..

[3] Naresh Kumar A., Murugan K., Madhiyazhagan P. (2013). Integration of botanicals and microbials for management of crop and human pests. Parasitol. Res..

[4] Zhao R. (2023). Identification and control of common cotton pests. Hunan Agric..

[5] Bragard C., Dehnen-Schmutz K., Di Serio F., Gonthier P., Jacques M.A., Miret J.A.J., Justesen A.F., Magnusson C.S., Milonas P., EFSA Panel on Plant Health (PLH) (2019). Pest categorisation of *Spodoptera litura*. EFSA J..

[6] Damiri N., Pujiastuti Y., Mulawarman, Astuti D.T., Afriani S.R., Rahim S.E. (2022). Biological control agent for *Spodoptera litura* on vegetable plants. Biodivers. J. Biol. Divers..

[7] Senthil-Nathan S. (2015). A review of biopesticides and their mode of action against insect pests. Environmental Sustainability.

[8] Saraswathi S., Shoba E., Dhayalan A., Pradhan N., Sreeramulu A.K., Rama T., Manjulakumari D. (2023). Overview of Pest Status, Control Strategies for *Spodoptera litura* (Fab.): A Review. J. Biopestic..

[9] Thakur N., Sharma A., Kaur S., Ahluwalia K.K., Sidhu A.K., Kumar S., Rustagi S., Singh S., Rai A.K., Sheikh S. (2024). Insect pest *Spodoptera litura* (Fabricius) and its resistance against the chemical insecticides: A review. Plant Sci. Today.

[10] Song Y., Cang X., He W., Zhang H., Wu K. (2024). Migration Activity of *Spodoptera litura* (Lepidoptera: Noctuidae) between China and the South-Southeast Asian Region. Insects.

[11] Srivastava K., Sharma D., Anal A.K.D., Sharma S. (2018). Integrated management of *Spodoptera litura*: A review. J. Integr. Pest Manag..

[12] Ahmad M., Sayyed A.H., Saleem M.A., Ahmad M. (2008). Evidence for field-evolved resistance to newer insecticides in *Spodoptera litura* (Lepidoptera: Noctuidae) from Pakistan. Crop Prot..

[13] Su J., Lai T., Li J. (2012). Susceptibility of field populations of *Spodoptera litura* (Fabricius) (Lepidoptera: Noctuidae) in China to chlorantraniliprole and the activities of detoxification enzymes. Crop Prot..

[14] Tong H., Su Q., Zhou X., Bai L. (2013). Field resistance of *Spodoptera litura* (Lepidoptera: Noctuidae) to organophosphates, pyrethroids, carbamates and four newer chemistry insecticides in Hunan, China. J. Pest Sci..

[15] Hilliou F., Chertemps T., Maïbèche M., Le Goff G. (2021). Resistance in the genus *Spodoptera*: Key insect detoxification genes. Insects.

[16] Hou W.T., Staehelin C., Elzaki M.E.A., Hafeez M., Luo Y.S., Wang R.L. (2021). Functional analysis of CYP6AE68, a cytochrome P450 gene associated with indoxacarb resistance in *Spodoptera litura* (Lepidoptera: Noctuidae). Pestic. Biochem. Physiol..

[17] Shi Y., Li W., Zhou Y., Liao X., Shi L. (2022). Contribution of multiple overexpressed carboxylesterase genes to indoxacarb resistance in *Spodoptera litura*. Pest Manag. Sci..

[18] Wu J.L., Leung E.L.H., Zhou H., Liu L., Li N. (2013). Metabolite analysis of toosendanin by an ultra-high performance liquid chromatography-quadrupole-time of flight mass spectrometry technique. Molecules.

[19] Ren L., Zheng G., Chen B., He L., Liao Y., Chen B. (2020). Evaluation of ten botanical insecticides against the sweet potato weevil, *Cylas formicarius* (Fabricius, 1798) (Coleoptera: Brentidae). Afr. J. Agric. Res..

[20] Pang F., Gong H., Wang X., Fang Y., Huang L., Lin J. (2018). Synthesis of danpihenone hydrazone compounds and their antibacterial activity. Chem. Res. Appl..

[21] Jaoko V., Taning C.N.T., Backx S., Motti P., Mulatya J., Vandenabeele J., Magomere T., Olubayo F., Smagghe G., Werbrouck S.P. (2023). Bioactivity-guided isolation of toosendanin and salanninolide from *Melia volkensii* and their antifeedant activity against economically important insect pests. Crop Prot..

[22] Huang Y., Liu J., Pang T., Li L. (2017). Growth inhibitory and antifeedant effects of sublethal concentrations of toosendanin on the rotifer *Brachionus plicatilis*. Biomass Bioenergy.

[23] Li H., Ma Z., Zhang X., Zhou Y. (2019). Effect of droplet size on the deposition of toosendanin on three crops. Trop. Biol..

[24] Shi Y.L., Li M.F. (2007). Biological effects of toosendanin, a triterpenoid extracted from Chinese traditional medicine. Prog. Neurobiol..

[25] Shu B., Lin Y., Huang Y., Liu L., Cai X., Lin J., Zhang J. (2024). Characterization and transcriptomic analyses of the toxicity induced by toosendanin in *Spodoptera frugiprerda*. Gene.

[26] Yamanaka N., Rewitz K.F., O’Connor M.B. (2013). Ecdysone control of developmental transitions: Lessons from *Drosophila* research. Annu. Rev. Entomol..

[27] Schoonhoven L.M., Luo L.E. (1994). Multiple mode of action of the feeding deterrent, toosendanin, on the sense of taste in *Pieris brassicae* larvae. J. Comp. Physiol. A.

[28] Li H., Zhang J., Ma T., Li C., Ma Z., Zhang X. (2020). Acting target of toosendanin locates in the midgut epithelium cells of *Mythimna separate* Walker larvae (Lepidoptera: Noctuidae). Ecotoxicol. Environ. Saf..

[29] Lin Y., Huang Y., Liu J., Liu L., Cai X., Lin J., Shu B. (2023). Characterization of the physiological, histopathological, and gene expression alterations in *Spodoptera frugiperda* larval midguts affected by toosendanin exposure. Pestic. Biochem. Physiol..

[30] Zhou Y., Li H.G., Huang Q., Liang S.J., Huang Q.Y., Zuo M.T., Bao M.H., He B.S. (2025). Toosendanin inhibits the growth of *Spodoptera litura* by inducing metabolic dysfunction in the midgut. Pestic. Biochem. Physiol..

[31] Xu Y., Dong J., Xia G., Mao G., Lu W. (2025). Synergistic antifeedant effects of tea saponin mixed with toosendanin and azadirachtin against *Spodoptera litura*. J. Environ. Entomol..

[32] Ahmad S., Pardini R.S. (1990). Mechanisms for regulating oxygen toxicity in phytophagous insects. Free Radic. Biol. Med..

[33] Li X., Schuler M.A., Berenbaum M.R. (2007). Molecular mechanisms of metabolic resistance to synthetic and natural xenobiotics. Annu. Rev. Entomol..

[34] Senthil-Nathan S. (2013). Physiological and biochemical effect of neem and other Meliaceae plants secondary metabolites against Lepidopteran insects. Front. Physiol..

[35] Laptiev A.B., Maltsev V.K. (2023). Effectiveness and safety of pesticides in protecting sunflowers from pests. Russ. Agric. Sci..

[36] Pereira V., Castilho P.C., Pereira J.A.M. (2025). Analysis of the environmental impact of botanical pesticides in soil. Agriculture.

[37] Chang J., Wang C., Gong L., Zhang Y., Liang C., Liu H. (2023). An overview of *Fructus Meliae Toosendan*: Botany, traditional uses, phytochemistry, pharmacology and toxicology. Biomed. Pharmacother..

[38] Rajab M. (2024). In Silico Analysis of Limonoid-Based Antifeedants from *Melia volkensii* Targeting the Ryanodine Receptor in *Spodoptera frugiperda*. Sci. Phytochem..

[39] Isman M.B. (2020). Botanical insecticides in the 21st century—Fulfilling their promise?. Annu. Rev. Entomol..

[40] Pavela R., Maggi F., Iannarelli R., Benelli G. (2019). Plant extracts for developing mosquito larvicides: From laboratory to the field, with insights on the modes of action. Acta Trop..

[41] Lin M., Bi X., Zhou L., Huang J. (2022). Insecticidal triterpenes in Meliaceae: Plant species, molecules, and activities: Part II (*Cipadessa*, *Melia*). Int. J. Mol. Sci..

[42] Shi Y., Wang W., Xu K. (1981). Electrophysiological analysis of the presynaptic blocking effect of toosendanin. Acta Physiol. Sin..

[43] Shi Y., Wang W., Liao C., Zhao S. (1986). Observation of the inhibitory effect of toosendanin on the chemoreceptor potentials of *Mythimna separata* larvae. Acta Entomol. Sin..

[44] Zhang X. (1982). Effects of Meliaceae Plant Insecticides on Feeding Deterrence and Growth Inhibition of Several Insect Pests. Master’s Thesis.

[45] Strand M.A.S. (1972). Annotated Bibliography on the Role of Foliage-Feeding Insects in the Forest Ecosystem.

[46] Ahn S.J., Badenes-Pérez F.R., Heckel D.G. (2011). A host-plant specialist, *Helicoverpa assulta*, is more tolerant to capsaicin from *Capsicum annuum* than other noctuid species. J. Insect Physiol..

[47] Coggan N., Clissold F.J., Simpson S.J. (2011). Locusts Use Dynamic Thermoregulatory Behaviour to Optimize Nutritional Outcomes. Proc. R. Soc. B Biol. Sci..

[48] Xu Y.-J., Luo F., Gao Q., Shang Y., Wang C. (2015). Metabolomics reveals insect metabolic responses associated with fungal infection. Anal. Bioanal. Chem..

[49] Muhammad J. (2025). Biochemical and molecular profile alterations in *Locusta migratoria migratorioides* (Orthoptera: Acrididae) induced by the entomopathogenic fungi. J. Nat. Pestic. Res..

[50] Lin D.J., Fang Y., Li L.Y., Zhang L.Z., Gao S.J., Wang R., Wang J.D. (2022). The insecticidal effect of the botanical insecticide chlorogenic acid on *Mythimna separata* (Walker) is related to changes in *MsCY*P450 gene expression. Front. Plant Sci..

[51] Zhang X., Chiu S.F. (1992). Effects of toosendanin on several enzyme systems of the cabbage worm *Pieris rapae* L.. Acta Entomol. Sin..

[52] Li E., Wu H., Wang Z., Li K., Zhang S., Cao Y., Yin J. (2023). PI3K/Akt/CncC signaling pathway mediates the response to EPN-Bt infection in *Holotrichia parallela* larvae. Pest Manag. Sci..

[53] Liu L.H., Guan L., Wang S. (2015). Effect of Ethanol Extract of *Chelidonium majus* on Protective Enzyme Activity in *Pieris rapae* L.. Heilongjiang Agric. Sci..

