# Supplementary material for: The Inhibitory Effect of Toosendanin on the Growth and Development of Spodoptera litura
Source: Insects. 2026 Jul 16;17(7):732. doi: 10.3390/insects17070732 (PMC13411656; doi:10.3390/insects17070732)
Supplement: Supplementary file 1 [file insects-17-00732-s001.zip › insects-4397934-supplementary.pdf]

Supplementary Materials

1. Preparation of crude enzyme extracts

Fresh insect samples were rinsed, blotted dry, and accurately weighed. Tissues were homogenized in pre-chilled extraction buffer at a ratio of 1:10 (w/v; tissue mass in g to buffer volume in mL) under ice-cold conditions. The homogenate was centrifuged at 8,000 × g for 10 min at 4 °C. The resulting supernatant was collected as the crude enzyme extract and kept on ice for immediate analysis. All enzyme activities and protein concentrations were subsequently determined using this extract.

2. Determination of hydrogen peroxide (H_2O_2) content (POD-related assay)

Hydrogen peroxide content was determined using a micro-assay kit (ml076323) according to the manufacturer's instructions. The absorbance was measured at **415 nm** after preheating the microplate reader for 30 min and zeroing with distilled water.

The total reaction volume was 500 μL, including 250 μL crude enzyme extract, 25 μL reagent II, 50 μL reagent III, and 250 μL reagent IV. After thorough mixing, samples were incubated at 25 °C in the dark for 10 min, and absorbance was recorded at 415 nm. The change in absorbance (ΔA) was used for calculations.

A standard curve was constructed as: $y = 0.7344x + 0.0006$

H_2O_2 content was calculated as: H_2O_2 (μmol g^{-1}) = [(ΔA − 0.0006) / 0.7344 × V_total] / (V_sample × W). When ΔA > 1, samples were appropriately diluted and corrected by dilution factors.

3. Trypsin activity assay

Trypsin activity was measured using a spectrophotometric assay kit (ml016821) at 555 nm. The instrument was preheated for 30 min and zeroed with distilled water.

The reaction system consisted of 25 μL crude enzyme extract and 975 μL working solution. Absorbance was recorded at 1 min and 2 min, and ΔA was calculated.

One unit of trypsin activity was defined as the amount of enzyme causing a 1.0 decrease in absorbance at **555 nm** per minute per mg protein under assay conditions.

Trypsin activity was calculated as: Trypsin (U mg^{-1} protein) = 40 × ΔA × V total / (C protein × V sample × T); Samples exceeding ΔA > 0.5 were diluted and re-assayed.

4. Carboxylesterase (CarE) activity assay

Carboxylesterase activity was determined using a micro-method kit (ml076534) at **490 nm**. Reagents were pre-equilibrated at 25 °C for 30 min.

The reaction mixture included 20 μL crude enzyme extract, 180 μL reagent I, 20 μL reagent II, and 20 μL reagent III. Absorbance was recorded at 0 min and 3 min, and ΔA was calculated.

CarE activity was expressed as: CarE (U mg^{-1} protein) = 13.67 × ΔA × V total / (C protein × V sample × T)

5. Glutathione S-transferase (GST) activity assay

GST activity was measured using a micro-assay kit (ml076443) at **340 nm**. The reaction was incubated in the dark for 10 min at room temperature.

Absorbance was recorded at 0 min and 10 min, and ΔA was calculated.

GST activity was defined as the amount of enzyme catalyzing the conjugation of 1 μmol CDNB with reduced glutathione per minute.

GST activity was calculated as: GST (nmol min^{-1} mg^{-1} protein) = −230 × ΔA × V total / (C protein × V sample × T)

6. α-Amylase activity assay

α-Amylase activity was determined using a micro-assay kit (ml076677) at **540 nm**. Samples and reagents were pre-incubated at 40°C.

After enzymatic reaction, the reaction was terminated in a boiling water bath, and absorbance was measured. ΔA was calculated as A control – A sample.

A standard curve was fitted as: y = 2.7831x − 0.1778

α-Amylase activity was calculated as: α-AL (U g^{-1}) = [(ΔA + 0.1778) / 2.7831 × V total] / (V sample × W × T)

7. Peroxidase (POD) activity assay

POD activity was measured using a spectrophotometric kit (ml076323) at **470 nm**. The reaction system contained 50 μL crude enzyme extract and 950 μL working solution.

Absorbance was recorded at 1 min and 2 min, and ΔA was calculated.

One unit of POD activity was defined as a change in absorbance of 0.01 per minute.

POD activity was calculated as: POD (U mg^{-1} protein) = ΔA × V total × 2000 / (0.01 × V sample × C protein)

8. Catalase (CAT) activity assay

CAT activity was determined using a molybdate colorimetric kit (ml076329) at **240 nm**.

The reaction system contained 35 μL crude enzyme extract and 1 mL working solution.

Absorbance was recorded after 1 min reaction at room temperature.

CAT activity was calculated as: CAT (nmol min^{-1} mg^{-1} protein) = ΔA × V total × 10^4 / (V sample × T × C protein)

9. Lipase (LPS) activity assay

Lipase activity was measured using a micro-assay kit (ml106888) at **710 nm**.

After incubation and centrifugation, the supernatant was used for colorimetric determination. ΔA was calculated based on sample and blank readings.

Lipase activity was calculated as: LPS (μmol min^{-1} mg^{-1} protein) = −16 × (A sample – A blank) × V total / [(A standard – A blank) × C_protein × V_sample × T]

10. Total superoxide dismutase (T-SOD) activity assay

T-SOD activity was measured using a commercial kit (A001-1-2) at **550 nm**.

The total reaction system (3.35 mL) was incubated at 37 °C for 40 min in the dark. Absorbance was recorded after color development.

ΔA was calculated as A_control − A_sample.

T-SOD activity was calculated as: T-SOD (U mg^{-1} protein) = (ΔA / A_control) / 50% × 3.35 / 0.05 / C_protein

11. Soluble protein determination (BCA method)

Soluble protein content was determined using a BCA protein assay kit (ml016897) at **562 nm**.

The reaction system contained 20 μL crude enzyme extract and 180 μL freshly prepared BCA working solution. After incubation at 37 °C for 30 min in the dark, absorbance was measured.

Protein concentration was calculated using a standard curve: y = ax + b

C protein (mg mL^{-1}) = (ΔA − b) / a × dilution factor

Protein content was used to normalize all enzyme activities.

The means, standard deviations, t-values, degrees of freedom, and significance levels (*p*-values; two-tailed) are presented in the table below. Levene's test for equality of variances showed that for both comparisons (12 h and 24 h), the F-value was 0.000 and the corresponding p-value was 1.000, indicating homogeneity of variances. Therefore, the t-test results assuming equal variances were used.

Table S1 Effect of toosendanin on the feeding preference of *Spodoptera litura* larvae

Treatment time	Toosendanin concentration (μg/g)	N	Mean	SD	*t* value	df	*p*-value (two-tailed)
12-h	0	3	49.33	2.31	4.243	4	0.013
12-h	25	3	41.33	2.31			
24-h	0	3	49.33	2.31	7.778	4	0.001
24-h	25	3	34.67	2.31			

Note: Levene's test for equality of variances showed that variances were equal in both comparisons (F = 0.000, p = 1.000), thus the t-test results assuming equal variances were used.

The means, standard deviations, significance levels, F-values, p-values, and degrees of freedom are presented in the table below. Different lowercase letters indicate significant differences among different concentration treatments ($p < 0.05$): Duncan's multiple range test was used when variances were homogeneous, and Tamhane's T2 test was used when variances were heterogeneous. In the data table, df1 represents the between-group degrees of freedom, and df2 represents the within-group degrees of freedom. In the table below, a p-value of 0.000 indicates $p < 0.001$.

Table S2-I Effect of toosendanin on maximum larval weight (g) and instar stage of *S. litura* on day 12 post-treatment

Treatment time	Toosendanin concentration (μg/g)	Levene's Test(*P*-value)	mean (g)	Standard deviation	Significance ($P < 0.05$)	F-value	df1	df2
1 d	0	.171	.117400	.0020000	a	964.298	5	12
	6.25	.171	.114133	.0007234	b	964.298	5	12
	12.5	.171	.110233	.0001155	c	964.298	5	12
	25	.171	.108100	.0007000	d	964.298	5	12
	50	.171	.098633	.0006506	e	964.298	5	12
	100	.171	.064000	.0013454	f	964.298	5	12
2 d	0	.084	.156767	.0057709	a	194.161	5	12
	6.25	.084	.151200	.0012288	b	194.161	5	12
	12.5	.084	.144700	.0031097	c	194.161	5	12
	25	.084	.139533	.0004041	c	194.161	5	12
	50	.084	.116000	.0025865	d	194.161	5	12
	100	.084	.094700	.0010440	e	194.161	5	12

Table S2-I (Continued) Effect of toosendanin on maximum larval weight (g) and instar stage of *S. litura* on day 12 post-treatment

3 d	0	.012	.461733	.0116646	a	832.326	5	12
	6.25	.012	.401767	.0050213	b	832.326	5	12
	12.5	.012	.341667	.0037528	c	832.326	5	12
	25	.012	.313933	.0034588	d	832.326	5	12
	50	.012	.273567	.0086286	e	832.326	5	12
	100	.012	.143400	.0005000	f	832.326	5	12
4 d	0	.179	.117400	.0020000	a	964.298	5	12
	6.25	.179	.114133	.0007234	b	964.298	5	12
	12.5	.179	.110233	.0001155	c	964.298	5	12
	25	.179	.108100	.0007000	d	964.298	5	12
	50	.179	.098633	.0006506	e	964.298	5	12
	100	.179	.064000	.0013454	f	964.298	5	12
5 d	0	.043	.735867	.0121533	a	2098.140	5	12
	6.25	.043	.647833	.0059138	b	2098.140	5	12
	12.5	.043	.595533	.0055717	c	2098.140	5	12
	25	.043	.535767	.0031723	d	2098.140	5	12
	50	.043	.448500	.0021284	e	2098.140	5	12
	100	.043	.266633	.0023116	f	2098.140	5	12
6 d	0	.022	2.364367	.0677211	a	1353.691	5	12
	6.25	.022	1.989533	.0082203	b	1353.691	5	12
	12.5	.022	1.912300	.0266513	c	1353.691	5	12
	25	.022	1.470200	.0154619	d	1353.691	5	12
	50	.022	1.304600	.0132774	e	1353.691	5	12
	100	.022	.484400	.0046357	f	1353.691	5	12

Table S2-I (Continued) Effect of toosendanin on maximum larval weight (g) and instar stage of *S. litura* on day 12 post-treatment

7 d	0	.117	2.775900	.0126961	a	2684.555	5	12
	6.25	.117	2.678533	.0371207	b	2684.555	5	12
	12.5	.117	2.594133	.0397945	c	2684.555	5	12
	25	.117	2.482500	.0142454	d	2684.555	5	12
	50	.117	2.156700	.0148970	e	2684.555	5	12
	100	.117	.830933	.0035921	f	2684.555	5	12
8 d	0	.003	4.125767	.0070437	a	1916.264	5	12
	6.25	.003	3.791933	.0261558	b	1916.264	5	12
	12.5	.003	3.354867	.0346839	c	1916.264	5	12
	25	.003	3.017367	.0851508	cd	1916.264	5	12
	50	.003	2.651867	.0154384	d	1916.264	5	12
	100	.003	1.296933	.0054151	e	1916.264	5	12
9 d	0	.002	7.488000	.0480209	a	2686.349	5	12
	6.25	.002	6.797733	.1591824	ab	2686.349	5	12
	12.5	.002	5.967100	.0645288	b	2686.349	5	12
	25	.002	4.826833	.0204121	c	2686.349	5	12
	50	.002	3.187033	.0039577	d	2686.349	5	12
	100	.002	1.763833	.0108583	e	2686.349	5	12
10 d	0	.046	10.088433	.0432851	a	1952.490	5	12
	6.25	.046	9.922900	.0696506	a	1952.490	5	12
	12.5	.046	9.405900	.2748195	ab	1952.490	5	12
	25	.046	7.874733	.0428838	b	1952.490	5	12
	50	.046	4.897633	.0633831	c	1952.490	5	12
	100	.046	2.306167	.0646031	d	1952.490	5	12

Table S2-I (Continued) Effect of toosendanin on maximum larval weight (g) and instar stage of *S. litura* on day 12 post-treatment

11 d	0	.099	12.453733	.0334315	a	3497.506	5	12
	6.25	.099	10.926567	.1545539	b	3497.506	5	12
	12.5	.099	10.671800	.1183839	c	3497.506	5	12
	25	.099	10.305700	.0734770	d	3497.506	5	12
	50	.099	8.059233	.0495105	e	3497.506	5	12
	100	.099	3.628167	.0612867	f	3497.506	5	12
12 d	0	.054	6.327533	.0390980	a	2373.445	5	12
	6.25	.054	5.790800	.0350056	b	2373.445	5	12
	12.5	.054	6.518300	.0630070	c	2373.445	5	12
	25	.054	7.952333	.1209100	d	2373.445	5	12
	50	.054	9.856267	.0473585	e	2373.445	5	12
	100	.054	4.720967	.0334965	f	2373.445	5	12

Table S2-II Effects of different concentrations of toosendanin on the maximum larval selected individuals per replicate (mg) of *S. litura*

Treatment time	Toosendanin concentration (μg/g)	Levene's Test(P-value)	mean (mg)	Standard deviation	Significance ($P < 0.05$)	F-value	df1	df2
11 d	0	.058	1245.3733	3.34315	a	413.187	5	12
11 d	6.25	.058	1092.6567	15.45539	b	413.187	5	12
11 d	12.5	.058	1067.1800	11.83839	c	413.187	5	12
11 d	25	.058	1030.5700	7.34770	d	413.187	5	12
12 d	50	.058	985.6267	4.73585	e	413.187	5	12
15 d	100	.058	942.9833	4.18867	f	413.187	5	12

Table S3-I Effect of toosendanin on individual pupal weight per replicate (g) of *S. litura*

Toosendanin concentration (μg/g)	Levene's Test(*P*-value)	mean (g)	Standard deviation	Significance ($P < 0.05$)	F-value	df1	df2
0	.002	.4573367	.00366286	a	389.593	5	12
6.25	.002	.4455933	.00096986	a	389.593	5	12
12.5	.002	.4388900	.00025942	a	389.593	5	12
25	.002	.4232300	.00046033	b	389.593	5	12
50	.002	.4170400	.00079542	c	389.593	5	12
100	.002	.4088067	.00074594	d	389.593	5	12

Table S4 GLM analysis of developmental responses of *S. litura* exposed toTSN

Response variable	β (dose)	SE	z value	p value	Odds Ratio (OR)	Pseudo R^2
Pupation percent	−0.0205	0.003	−7.820	<0.001	0.9797	0.9662
Adult emergence percent	−0.0141	0.003	−5.402	<0.001	0.9860	0.7892
Pupal deformity percent	0.0249	0.003	8.803	<0.001	1.0252	0.9873
Adult deformity percent	0.0173	0.004	4.851	<0.001	1.0174	0.7013

Data were analyzed using a binomial GLM with a logit link function. β represents the regression coefficient for TSN concentration. SE: standard error. OR: odds ratio per unit increase in dose. Pseudo R^2: Cox–Snell coefficient of determination. All effects were statistically significant at $P < 0.001$.

The means (mean-ind.), standard deviations, F-values, between-group degrees of freedom (df1), and within-group degrees of freedom (df2) are presented in the table below.

Table S3-II Effect of toosendanin at different concentrations on the number of pupated individuals of *S. litura*

Treatment time	Toosendanin concentration (μg/g)	mean (ind.)	Standard deviation	F-value	df1	df2
12 d	0	2.33	.577	49.000	5	12
	6.25	0.00	0.000	49.000	5	12
	12.5	0.00	0.000	49.000	5	12
	25	0.00	0.000	49.000	5	12
	50	0.00	0.000	49.000	5	12
	100	0.00	0.000	49.000	5	12
13 d	0	7.00	1.000	210.225	5	12
	6.25	15.00	1.000	210.225	5	12
	12.5	5.33	.577	210.225	5	12
	25	3.67	.577	210.225	5	12
	50	0.00	0.000	210.225	5	12
	100	0.00	0.000	210.225	5	12
14 d	0	29.00	1.000	551.467	5	12
	6.25	24.33	.577	551.467	5	12
	12.5	27.33	1.528	551.467	5	12
	25	22.00	1.000	551.467	5	12
	50	4.67	.577	551.467	5	12
	100	0.00	0.000	551.467	5	12
15 d	0	11.00	1.000	200.640	5	12
	6.25	8.67	.577	200.640	5	12
	12.5	7.33	1.155	200.640	5	12
	25	12.67	.577	200.640	5	12
	50	18.33	.577	200.640	5	12
	100	0.00	0.000	200.640	5	12
16 d	0	0.00	0.000	333.400	5	12
	6.25	0.00	0.000	333.400	5	12
	12.5	5.33	.577	333.400	5	12
	25	4.33	.577	333.400	5	12
	50	13.33	.577	333.400	5	12
	100	2.33	.577	333.400	5	12

Table S3-II (Continued) Effect of toosendanin at different concentrations on the number of pupated individuals of *S. litura*

17 d	0	0.00	0.000	12.264	5	12
	6.25	0.00	0.000	12.264	5	12
	12.5	0.00	0.000	12.264	5	12
	25	1.33	2.309	12.264	5	12
	50	4.00	1.000	12.264	5	12
	100	5.00	1.000	12.264	5	12
18 d	0	0.00	0.000	1225.000	5	12
	6.25	0.00	0.000	1225.000	5	12
	12.5	0.00	0.000	1225.000	5	12
	25	0.00	0.000	1225.000	5	12
	50	0.00	0.000	1225.000	5	12
	100	11.67	.577	1225.000	5	12
19 d	0	0.00	0.000	363.000	5	12
	6.25	0.00	0.000	363.000	5	12
	12.5	0.00	0.000	363.000	5	12
	25	0.00	0.000	363.000	5	12
	50	0.00	0.000	363.000	5	12
	100	11.00	1.000	363.000	5	12
20 d	0	0.00	0.000	48.000	5	12
	6.25	0.00	0.000	48.000	5	12
	12.5	0.00	0.000	48.000	5	12
	25	0.00	0.000	48.000	5	12
	50	0.00	0.000	48.000	5	12
	100	4.00	1.000	48.000	5	12
21 d	0	0.00	0.000	49.000	5	12
	6.25	0.00	0.000	49.000	5	12
	12.5	0.00	0.000	49.000	5	12
	25	0.00	0.000	49.000	5	12
	50	0.00	0.000	49.000	5	12
	100	2.33	.577	49.000	5	12

Data are presented as mean ± standard deviation (SD). Statistical analysis was performed using two-way ANOVA (Treatment × Time) followed by Bonferroni post hoc correction. Significant differences were denoted by letter grouping: lowercase letters indicate significant differences among time points within the same treatment, while uppercase letters indicate significant differences among treatments within the same time point ($P < 0.05$).

Table S5-I. Effects of TSN on enzyme activities of *Spodoptera litura* larvae based on two-way ANOVA (Treatment × Time)

Enzyme	Factor	F value	df	p value	Partial η²
Lipase	Treatment	26.493	1,12	<0.001	0.688
	Time	35.133	2,12	<0.001	0.854
	Treatment × Time	0.992	2,12	0.399	0.142
Trypsin	Treatment	188.674	1,12	<0.001	0.940
	Time	94.865	2,12	<0.001	0.941
	Treatment × Time	61.386	2,12	<0.001	0.911
α-Amylase	Treatment	1068.905	1,12	<0.001	0.989
	Time	313.679	2,12	<0.001	0.981
	Treatment × Time	94.473	2,12	<0.001	0.940

Table S5-II Descriptive statistics (mean ± SD) of lipase activity in *S. litura* under different treatments and exposure times

Treatment	Lipase activity (U mg⁻¹ protein)		
	24 h	48 h	72 h
Control	3.457 ± 0.105 bA	4.268 ± 0.193 aA	3.940 ± 0.208 aA
50μg/g	3.148 ± 0.179 bA	3.930 ± 0.138 aA	3.386 ± 0.143 bB

Table S5-III Descriptive statistics (mean ± SD) of trypsin activity in *S. litura* under different treatments and exposure times

Treatment	Trypsin activity (U mg⁻¹ protein)		
	24 h	48 h	72 h
Control	2.170 ± 0.058 aA	2.518 ± 0.349 aA	2.220 ± 0.189 aA
50μg/g	2.411 ± 0.243 cA	5.745 ± 0.332 aB	3.391 ± 0.113 bB

Table S5-IV Descriptive statistics (mean ± SD) of *α*-Amylase activity in *S. litura* under different treatments and exposure times

Treatment	*α*-Amylase activity (U mg⁻¹ protein)		
	24 h	48 h	72 h
Control	0.130 ± 0.003 bA	0.167 ± 0.003 aA	0.133 ± 0.004 bA
50μg/g	0.197 ± 0.011 bB	0.321 ± 0.006 aB	0.202 ± 0.008 bB

Table S5-V Detoxification statistics (mean ± SD) of CYP450 activity in *S. litura* under different treatments and exposure times

Treatment	CYP450 activity (U mg⁻¹ protein)		
	24 h	48 h	72 h
Control	0.021 ± 0.001 aA	0.014 ± 0.001 bA	0.013 ± 0.001 cA
50μg/g	0.023 ± 0.001 aB	0.015 ± 0.001 bA	0.007 ± 0.001 cB

Table S5-VI Detoxification statistics (mean ± SD) of Glutathione S-transferase activity in *S. litura* under different treatments and exposure times

Treatment	Glutathione S-transferase activity (U mg⁻¹ protein)		
	24 h	48 h	72 h
Control	2.290 ± 0.206 aA	4.132 ± 1.794 bA	2.806 ± 0.657 aA
50μg/g	2.290 ± 0.206 bA	4.132 ± 1.794 aB	2.806 ± 0.657 bB

Table S5-VII Detoxification statistics (mean ± SD) of CarE in *S. litura* under different treatments and exposure times

Treatment	CarE activity (U mg⁻¹ protein)		
	24 h	48 h	72 h
Control	2.170 ± 0.058 bA	2.518 ± 0.349 bA	2.220 ± 0.189 aA
50μg/g	2.411 ± 0.243 cB	5.745 ± 0.332 aB	3.391 ± 0.113 bA

Table S5-VIII Oxidative stress enzyme statistics (mean ± SD) of Superoxide dismutase (SOD) activity in *S. litura* under different treatments and exposure times

Treatment	Superoxide dismutase (SOD) activity (U mg⁻¹ protein)		
	24 h	48 h	72 h
Control	18.943 ± 1.011 aA	12.838 ± 1.336 bA	12.558 ± 2.173 bA
50μg/g	25.181 ± 0.306 bB	33.443 ± 0.657 aB	21.768 ± 1.030 cB

Table S5-IX Oxidative stress enzyme statistics (mean ± SD) of Peroxidase activity in *S. litura* under different treatments and exposure times

Treatment	Peroxidase activity (U mg⁻¹ protein)		
	24 h	48 h	72 h
Control	81.164 ± 5.418 abA	70.999 ± 10.471 bA	94.405 ± 9.476 aA
50μg/g	82.660 ± 9.065 abA	189.871 ± 7.236 aB	157.322 ± 12.108 bB

Table S5-X Oxidative stress enzyme statistics (mean ± SD) of Catalase (CAT) activity in *S. litura* under different treatments and exposure times

Treatment	Catalase (CAT) activity (U mg⁻¹ protein)		
	24 h	48 h	72 h
Control	26.957 ± 2.9808 aA	47.244 ± 1.187 bA	42.850 ± 3.163 bA
50μg/g	54.220 ± 4.526 bB	78.626 ± 5.583 aB	54.380 ± 6.928 bA
